# Factors associated with health-related quality of life in a cohort of cancer survivors in New Jersey

**DOI:** 10.1186/s12885-023-11098-5

**Published:** 2023-07-14

**Authors:** Sharon Manne, Katie Devine, Shawna Hudson, Deborah Kashy, Denalee O’Malley, Lisa E. Paddock, Elisa V. Bandera, Adana A. M. Llanos, Angela Fong, Neetu Singh, Sara Frederick, Andrew M. Evens

**Affiliations:** 1 Rutgers Cancer Institute of New Jersey, New Brunswick, NJ USA; 2 Department of Family Medicine and Community Health, Rutgers Robert Wood Johnson Medical School, New Brunswick, NJ USA; 3 Department of Psychology, College of Social Science, Michigan State University, East Lansing, MI USA; 4 Cancer Surveillance Research Program, Cancer Epidemiology Services, Department of Health, Rutgers Cancer Institute of New Jersey, New Jersey State Cancer Registry, New Brunswick, Trenton, New Jersey USA; 5 Mailman School of Public Health, Columbia University, New York, NY USA

**Keywords:** Cancer survivorship, Quality of life, Social determinants, Unmet needs

## Abstract

**Background:**

Although there is extensive literature on correlates of health-related quality of life (HRQoL) among cancer survivors, there has been less attention paid to the role of socioeconomic disadvantage and survivorship care transition experiences in HRQoL. There are few large cohort studies that include a comprehensive set of correlates to obtain a full picture of what is associated with survivors’ HRQ0L. This cohort study of recent cancer survivors in New Jersey aimed to explore the association between social determinants of health, health history, health behaviors, survivorship care experiences, and psychosocial factors in HRQoL.

**Methods:**

Eligible survivors were residents of New Jersey diagnosed with genitourinary, female breast, gynecologic, colorectal, lung, melanoma, or thyroid cancers. Participants completed measures of social determinants, health behaviors, survivorship care experiences, psychosocial factors, and HRQoL. Separate multiple regression models predicting HRQoL were conducted for each of the five domains (social determinants, health history, health behaviors, survivorship care experiences, psychosocial factors). Variables attaining statistical significance were included in a hierarchical multiple regression arranged by the five domains.

**Results:**

864 cancer survivors completed the survey. Lower global HRQoL was associated with being unemployed, more comorbidities, a less healthy diet, lower preparedness for survivorship, more unmet support needs, and higher fear about cancer recurrence. Two psychosocial factors, unmet support needs and fear of recurrence, played the most important role in HRQoL, accounting for more than 20% of the variance. Both unmet support needs and fear of recurrence were significant correlates of physical, functional, and emotional HRQoL domains.

**Conclusions:**

Interventions seeking to improve cancer survivors’ HRQoL may benefit from improving coordinated management of comorbid medical problems, fostering a healthier diet, addressing unmet support needs, and reducing survivors’ fears about cancer recurrence.

**Supplementary Information:**

The online version contains supplementary material available at 10.1186/s12885-023-11098-5.

## Introduction

Due to improvements in effective treatments for many cancers, the population of cancer survivors is growing rapidly. There are approximately 18 million cancer survivors in the United States, and it is estimated that there will be approximately 27 million survivors by 2050 [1]. It is well-documented that cancer survivors experience challenges and issues which can develop and/or persist throughout the survivorship trajectory and ultimately impact survivors’ health-related quality of life (HRQOL) [2–6].

Contributing factors for lower HRQOL can be categorized into key domains. One key domain is *social determinants of health*, which include age, race/ethnicity, sex, education, employment status, income, and financial hardship. Although results vary across studies depending on cancer type, social determinants for lower HRQOL include female gender identity, [6, 7] younger age, [2, 8] being unmarried, [7] unemployed, [9] and uninsured, [9] having lower income, [2] and less education, [2] and being a racial or ethnic minority [2, 7–14]. In addition, recent evidence suggests that greater cancer-related financial hardship [11, 12] is associated with lower HRQOL. A second key domain is *health history*, which includes both cancer-related variables such as cancer type, cancer stage, time since diagnosis, and other health concerns such as medical comorbidities and obesity. Later stage, a longer time since diagnosis, more medical comorbidities, [2, 15, 16] and obesity [2, 17] are associated with lower HRQoL. A third domain is *lifestyle factors*, which includes physical activity, tobacco and alcohol use, and healthy dietary practices. Lower levels of physical activity, [2, 18–21] tobacco and alcohol use, [22–25] and poor dietary practices [19] have been consistently associated with lower HRQoL. A fourth domain is *psychosocial factors*. More unmet cancer-related support needs [26] and higher fear of recurrence [27] have been associated with lower HRQoL.

A less understood but likely important risk factor in survivors’ HRQoL is the survivor’s oncology care transition experiences, which ***was*** defined as how the oncology care team manages survivor’s transition off of active treatment. As unmet cancer-related information, social, and psychological support needs have been associated with HRQoL, [26, 28–30] providers’ discussions about these needs has the potential to improve HRQoL.

### Study background and aims

Although there is an extensive literature on correlates of HRQoL, there has been less attention paid to the role of socioeconomic disadvantage and survivorship care transition experiences in HRQoL. In addition, there are few studies that have included a comprehensive set of potential correlates of quality of life. There have been no survivorship HRQoOL studies using a statewide sample with a broad set of potential correlates. With more than 543,000 cancer survivors, [1] NJ is one of the most densely populated and diverse populations in the United States. The state has a high proportion of racial and ethnic minority and/or immigrant residents, and there are large income disparities across the state. The population density in counties close to New York City is higher than national averages, but the states’ southern and northwestern counties have lower population densities. The statewide sample offers the opportunity to evaluate the relative contribution of a comprehensive set of risk factors for poor HRQoOL in a large, representative sample. In addition, we are examining little-studied but modifiable risk factors such as preparedness for survivorship and survivorship care practices.

The study’s aim as to examine the association between social determinants of health, health history, health behaviors, survivorship care experiences, and psychosocial factors in the HRQoL among a sample of recent cancer survivors diagnosed in New Jersey (NJ). Recent was defined as between approximately two and five years before study participation). HRQoL ***was*** defined as the domains of quality of life that are directly affected by cancer and its treatment. HRQoL is multi-dimensional construct and includes physical, psychological, functional, and social domains related to a person’s perception of quality of life affected by health status.

## Methods

The methods and measures have been described in previous work published from this study [31].

### Eligibility

Eligibility criteria included individuals who were: (a) 18–85 years of age; (b) a current resident of NJ; (c) diagnosed in 2015, 2016, or 2018 with a primary case of genitourinary (i.e., bladder and prostate), female breast, gynecologic (i.e., cervical, endometrial, ovarian), colorectal, lung, melanoma, or thyroid cancer and; (d) able to read and speak English.

The New Jersey State Cancer Registry (NJSCR), a high-quality population-based cancer registry that has been in existence since 1979, was used to identify eligible cases. To create a sample that is representative of New Jersey’s cancer population, proportional stratified random sampling was used to select cases using the following strata: county, race, ethnicity, gender, and cancer type. Races other than white and Hispanic ethnicity were oversampled to account for typically lower participation in these populations. Counties were grouped into regions for further analysis: North NJ (Bergen, Essex, Hudson, Morris, Passaic, Sussex, Warren); Central NJ (Hunterdon, Mercer, Middlesex, Monmouth, Ocean, Somerset, Union); and South NJ (Atlantic, Burlington, Camden, Cape May, Cumberland, Gloucester, and Salem counties).

### Procedures and participation

The study received institutional review board approval from the Rutgers Institutional Review Board prior to study commencement and conforms to recognized standards of United States Federal Policy for the Protection of Human Subjects. After potentially eligible participants were identified through the NJSCR, eligibility was confirmed by a Certified Tumor Registrar. NJSCR staff mailed a recruitment package, which included a cover letter, study information, questionnaire, and postage-paid return envelope, to prospective participants. One week after the information package was mailed, individuals were called by NJSCR staff to confirm receipt of the recruitment package and to answer any questions about the study. Interested patients completed the questionnaire and mailed it back to the host institution. Prospective participants were called 1–2 times per week at varying days and times to increase patient contact and participation, with a maximum of 8 calls; those who could not be contacted were considered passive refusers. When reached, individuals who did not agree to participate were considered active refusers. In addition to follow-up phone calls, recruitment packets were resent up to three times during the recruitment period. A returned, completed questionnaire was considered to be an individual’s consent for study inclusion per Rutgers Institutional Review Board written informed consent waiver approval. Participants received a $25 gift card as an incentive [31].

Between August 2018 and January 2022, 3,348 individuals met the initial eligibility requirements and were contacted for study participation. Among them, 538 were deemed ineligible, 1,830 refused, 116 were unable to be contacted (could not locate correct address or phone), and 864 returned the survey ***(32.1%*** response rate). Comparisons of the 864 acceptors and the 1830 refusers based on available data (sex, race, ethnicity, age, cancer type, cancer stage) indicated only two differences: Hispanic survivors were more likely to decline participation (75.5%) than non-Hispanic survivors (67.5%) (Chi-square = 4.4, *p* < .05) and there were significant differences based on type of cancer (Chi-square = 20.4, *p* < .001), with breast cancer survivors having the lowest refusal rate (61.8%) and thyroid cancer survivors having the highest refusal rate (74.8%).

### Measures

#### HRQoL

The Functional Assessment of Cancer Therapy- General (FACT-G) (Version 4) [32] is a widely-used patient-reported outcome instrument which assesses HRQoL in cancer patients. The survey has four subscale domains: physical well-being (PWB, 7 items), social/family well-being (SWB, 7 items), emotional well-being (EWB, 7 items), and functional well-being (FWB, 7 items). Likert ratings range from *not at all* (0) to *very much* (4). Averages were computed for scale scores. Reliability for total HRQoL α = 0.92, and subscale reliabilities were: PWB α = 0.87, SWB α = 0.85, EWB α = 0.70, and FWB α = 0.92.

#### Social determinants of health

Demographics. Variables included age, biological sex, race, ethnicity, marital status, and nativity. Race and ethnicity were coded as 1 = White, not Hispanic and 0 = other. Education was coded 1 = Bachelor’s degree or more, 0 = less than Bachelor’s degree. Employment status was coded 1 = employed, 0 = unemployed/disabled/retired = 0. Nativity was coded as US born or not.

Financial hardship. Fifteen items assessed cancer-related financial hardships including: borrowing money/go into debt, could not afford medications, declaring bankruptcy, unable to cover cost of care visit, and worry about paying bills (*yes/no*) [33, 34]. Because this variable was highly skewed, for this analysis, the scale was dichotomized to 0 and 1.

Neighborhood socioeconomic disadvantage. The Area Deprivation Index (ADI) is based on a measure originally created by the Health Resources & Services Administration (HRSA) that has been adapted and validated to the Census Block Group neighborhood level [35, 36]. The ADI allows for rankings of neighborhoods by socioeconomic disadvantage in a region of interest [37]. It includes factors for the domains of income, education, employment, and housing quality. To calculate an ADI, census data block group data are ranked in percentiles from 1 to 100 (1 = lowest disadvantage within the nation to 100 = highest level of disadvantage.

#### Health history

Cancer history. Self-reported cancer-related medical variables were: Cancer site, diagnosis, diagnosis date (to calculate time since diagnosis), and treatments received (surgery, chemotherapy, radiation). Cancer stage was provided by the NJSCR.

Comorbidities. A checklist of 23 health conditions derived from the Health Information National Trends Survey (HINTS) was used. Responses included yes or no [38, 39]. Frequency of yes responses were tallied to score the measure.

#### Health behaviors

Current alcohol use. One item from the Follow-up Care Use Health Outcomes Survey (FOCUS) questionnaire [40] was used to assess alcohol consumption [40]: “Have you had any beer, wine, wine coolers, mixed drinks, liquor, or other alcoholic beverages during the past month?” (*yes, no, don’t know, prefer not to answer*).

Tobacco use. Tobacco use was assessed using a single item to assess current smoking from the Follow-up Care Use and Health Outcomes Survey (FOCUS) questionnaire^40^ (*every day, some days, not at all)* Use was categorized as yes/no.

Physical activity. Physical activity was measured using the Godin Leisure Time Exercise Questionnaire [41]. Four items measure assess the number of sessions per week when participant engages in 30 min or more of mild, moderate, and strenuous activity. Scores below 14 were classified as insufficiently active/sedentary, scores from 14 to 23 were classified as moderately active, and scores of 24 or higher were classified as active.

Healthy diet. A **s**even item measure was developed based on the dietary practices recommended by World Cancer Research Fund/American Institute of Cancer Research [42]. Categories included fruits, vegetables, sugar-sweetened beverages, whole grain products, red meat, processed meat, dairy products, and fast foods (*never, once per week, 2–4 times per week, 5–6 times per week, once per day*, and *2 or more times per day*). The cancer-specific nutritional recommendations from the World Cancer Research Fund/American Institute of Cancer Research [42] were used for scoring. A higher score indicated closer alignment with the nutritional recommendations.

Obesity/BMI. Obesity was calculated using the body mass index (BMI), which was calculated from height and weight as weight in kg divided by the square of height.

#### Survivorship care experiences

Provider survivorship care practices. A single index was created from six items. Three items from the Follow-up Care Use and Health Outcomes Survey [40] assessed whether participants were provided: (1) a treatment summary; (2) instructions about follow-up appointments and who to see for routine cancer checkups; and (3) access to a patient navigator. Three items from the HINTS [39, 43] assessed whether a provider had ever discussed: (1) late or long-term side effects; (2) emotional or social needs related to cancer; and (3) lifestyle or health recommendations such as diet, weight control, exercise, or quitting smoking (yes/no). Items were summed.

Preparedness for survivorship. Eight items adapted from Manne and colleagues [44] assessed whether information received about survivorship was sufficient, easy to understand, helpful, addressed needs, addressed how to manage symptoms, and look for signs of cancer (1 = *strongly disagree*, 5 = s*trongly agree*). Higher scores indicate better preparedness; α = 0.90.

Information needs. A 12-item scale was adapted from the Information about Health-related Topics section of FOCUS [34, 40] and existing literature. Topics included cancer-related follow-up tests, symptoms that should prompt calling a doctor, late and long-term side effects, managing anxiety, insurance, maintaining physical health, financial issues, and managing symptoms (*yes*/*no*). α = 0.91.

#### Psychosocial factors

Unmet supportive care needs. The 35-item Supportive Care Needs Survey [45] assessed needs across physical, psychological, health care systems and information, patient care and support, sexuality, and financial domains (*Met need, Unmet need, no need*). An unmet needs score was calculated.

Fear of recurrence. Participants completed the Concerns about Recurrence Scale, a 4-item scale assessing worries about the possibility of cancer recurrence (1 = *not at all*; 6 = *extremely)*. Higher scores indicated greater fear. α = 0.93.

### Data analytic approach

Given the large number of possible predictors, we took a two-step analytic approach. First, we conducted five separate multiple regression models predicting HRQOL in which all variables within a particular domain (e.g., all sociodemographic or all health history variables) were treated as predictors. Results from these analyses, along with descriptive statistics, are presented in Supplemental Tables 1–4. Using the results from these analyses we selected only those variables that attained p < .05 statistical significance to include in the primary analysis. The primary analysis was a hierarchical multiple regression arranged in the following steps: sociodemographic variables, health history variables, health behavior variables, survivorship care experiences, and psychosocial characteristics. Change in R^2^ was computed for each step. The same approach was used to predict the four subscales of HRQoL.

## Results

### Sample characteristics

**Table** 1 presents all variables included in the models. Eight hundred and sixty four survivors participated, ranging age from 20 to 87 years (M = 63.6 years). The majority of participants were White, non-Hispanic, US born, married, had completed at least a college-level education, and carried health insurance. The prevalence of cancer-related financial hardship was relatively low, and there was a relatively equal distribution of participants across the range of economic advantaged/disadvantaged areas of New Jersey.

**Table 1:** Descriptive Statistics for the Sample

	N (%)	M (SD)
**SOCIODEMOGRAPHICS**		
**Sex**		
Male	349 (40.4)	
Female	515 (59.6)	
**Age (years)**		64.4 (10.8)
**Race**		
White	693 (80.2)	
Black	111 (12.8)	
Asian or Asian American	48 (5.6)	
American Indian/Alaska native	2 (0.1)	
Other	11 (1.3)	
**Hispanic ethnicity**		
Yes	51 (5.9)	
No	813 (94.2)	
**US-born**		
Yes	737 (85.3)	
No	118 (13.7)	
Prefer not to answer	4 (0.5)	
**Marital status**		
Married	565 (67.2)	
Widowed	79 (9.0)	
Divorced/Separated	109 (12.6)	
Single	109 (12.7)	
Missing	14 (1.6)	
**Education**		
High school graduate or less	195 (22.6)	
Some college/Post high school	207 (24.0)	
College graduate	262 (30.3)	
Post college	185 (21.4)	
Missing	15 (3.4)	
**Employment status**		
Employed/homemaker/student/part-time	392 (45.4)	
Unemployed/retired/disabled/	449 (52.0)	
Missing	23 (2.7)	
**Household income**		
< $10,000	37 (4.3)	
$10,000 - $19,999	52 (6.0)	
$20,000 – $29,999	36 (4.2)	
$30,000 - $39,999	37 (4.3)	
$40,000 - $49,999	43 (5.0)	
$50,000 - $59,999	47 (5.4)	
$60,000 - $69,999	55 (6.4)	
$70,000- $79,999	43 (5.0)	
$80,000 -$89,999	46 (5.3)	
≥ $90,000	266 (30.8)	
Prefer not to answer/Missing	202 (23.4)	
**Health insurance status**		
Uninsured	12 (1.4)	
Insured	819 (94.7)	
Missing	33 (3.8)	
**Financial Hardship**		
0	565 (67.7)	
1	105 (12.2)	
2	2 (0.2)	
≥ 3	110 (12.7)	
Missing	3 (0.3)	
**Area Deprivation Index**		
1 (most deprived)	82 (9.5)	
2	86 (10.0)	
3	89 (10.3)	
4	102 (11.8)	
5	86 (10.0)	
6	87 (10.1)	
7	85 (9.8)	
8	64 (7.4)	
9	97 (11.2)	
10 (least deprived)	86 (10.0)	
**HEALTH HISTORY**		
**Primary cancer diagnosis**		
Bladder	82 (9.4)	
Breast	216 (25.0)	
Colorectal	99 (11.4)	
Prostate	154 (17.8)	
Gynecologic	88 (10.1)	
Lung	86 (10.0)	
Malignant skin	78 (9.9)	
Thyroid	62 (7.2)	
**Stage**		
0	61 (7.1)	
1	595 (68.9)	
2	51 (5.9)	
3	89 (10.3)	
4	43 (5.0)	
Missing	24 (2.8)	
**Surgery**		
Yes	723 (83.7)	
No	141 (16.3)	
**Chemotherapy**		
Yes	253 (29.3)	
No	611 (70.7)	
**Radiation**		
Yes	342 (39.6)	
No	506 (58.6)	
**Time since diagnosis (months)**		37.8 (7.2)
**Comorbidities**		
0	155 (17.9)	
1	217 (25.1)	
2	187 (21.6)	
>2	303 (35.1)	
Missing	2 (0.2)	
**BODY MASS INDEX (BMI) AND HEALTH BEHAVIORS**		
**BMI (kg/m** ^**2**^ **)**		28.8 (6.3)
Underweight	7 (0.8)	
Healthy weight	241 (27.9)	
Overweight	283 (32.8)	
Obese	286 (33.1)	
Missing	47 (5.4)	
**Physical activity**		
Insufficiently active/sedentary (LSI < 14)	259 (30.2)	
Moderately active (LSI = 14–23)	161 (18.8)	
Active (LSI ≥ 24)	379 (44.2)	
**Smoking status**		
Every day	46 (5.3)	
Some days	24 (5.7)	
Not at all	348 (83.3)	
Missing	445	
**Alcohol consumption past month**		
Yes	532 (61.6)	
No	298 (34.5)	
Prefer not to answer/Don’t know/Missing	34 (3.9)	
**Healthy diet intake**		4.5 (0.68)
**SURVIVORSHIP CARE EXPERIENCES**		
Preparedness		3.3 (0.81)
Care practices		3.7 (1.6)
Treatment summary provided (yes)	451 (52.2)	
Instructions about follow-up care (yes)	771 (89.2)	
Offered navigator (yes)	284 (32.9)	
Discussed late and long-term effects (yes)	565 (65.4)	
Discussed lifestyle (yes)	640 (74.0)	
Discussed emotional effects (yes)	500 (57.9)	
Information needs		4.3 (3.7)
**PSYCHOSOCIAL**		
Support Needs	4.1 (6.36)	
Fear of Recurrence	2.7 (0.68)	

**Note.** LSI = Leisure Survey Index

Participants were diagnosed with bladder (9.4%), breast (25%), prostate cancer (17.8%), colorectal (11.4%), gynecological (10%), lung (10%), melanoma (9.9), or thyroid cancers (7.2%). Seventy-six percent of participants were diagnosed with an early-stage cancer, and the average time since diagnosis was three years (range = 1.7–4.9). One-third had two or more comorbidities. Approximately 66% were overweight or obese, and about a third (30.2%) were insufficiently active. The vast majority (90.6%) did not currently smoke, but more than half (61.6%) had consumed alcohol in the last month. Healthy dietary practices were relatively high (*M* = 4.5 on a 6-point scale).

In terms of survivorship care experiences, the average levels of preparedness were high (*M* = 4.5 on a 6-point scale). Approximately half had received a treatment summary. The majority reported receiving instructions about recommended follow-up appointments (89.2%) and about late and long-term side effects (65.4%). Discussions about lifestyle and health behaviors (74.1%) and emotional and social needs related to cancer (57.8%) were relatively common. Only a third were offered a patient navigator during or after treatment. Information needs nominated most frequently were: “Cancer symptoms that should prompt you to call your doctor” (52.4%), “Medical advances in cancer treatment” (49.6%), and “Maintaining good physical health after cancer treatment” (47.5%) (data not shown) The most common unmet support needs were psychosocial: uncertainty about the future (24.5%) and fear of the cancer spreading (22.1%) (data not shown). Average levels of fear about cancer recurrence were midrange (*M* = 2.7, range, 1–6).

### Multiple regression results predicting overall HRQoL

Results of the five individual regression analyses that examined associations between all indicators within a domain (e.g., social determinants, health history) are presented in **Table** 2. In the first panel, of the nine social determinant variables, biological sex, employment status, financial hardship, and area deprivation were significant predictors of overall HRQoL. Men and employed survivors reported higher HRQoL. Survivors experiencing financial hardship and/or those living in an area with a higher ADI reported lower HRQoL. Among the health history variables, there were three significant predictors: Treatment with radiation, treatment with chemotherapy, and number of comorbidities. Survivors who were treated with radiation or chemotherapy and those with higher numbers of comorbidities reported significantly lower HRQoL. Of the five health behavior variables, all but BMI were significant predictors of HRQoL. Current smokers reported lower HRQoL, but individuals who had used alcohol in the past month, who had higher physical activity, and who ate healthier diets had higher HRQoL. Of the three survivorship care experience variables, survivors with higher information needs reported lower HRQoL. Survivors with higher levels of preparedness reported higher HRQoL. Finally, both psychosocial predictors were significant predictors of HRQoL. Survivors reporting more unmet support needs and higher fear of cancer recurrence reported lower HRQoL.

**Table 2:** Five sets of regression results predicting overall HRQoL separately with the full set of predictors within a type as well as descriptive statistics for those predictors

	Regression Results				Descriptive Statistics			
Sociodemographic regression	*b*	*Β*	*t*	*P*	*M*	*SD*	*N*	*%*
Biological Sex: Male	0.089	0.074	2.08	0.038			349	40.4
White-Not Hispanic	0.016	0.012	0.30	0.763			645	76.2
Married	0.014	0.011	0.31	0.758			565	66.5
Education: BA or more	0.033	0.028	0.78	0.437			447	52.7
Employed	0.206	0.174	4.21	0.000			392	46.6
US Born	0.019	0.011	0.29	0.770			737	86.2
Any Financial Hardship	− 0.336	− 0.263	-7.36	0.000			276	32.1
Age	0.004	0.079	1.84	0.066	64.39	10.76		
Area Deprivation Index	− 0.002	− 0.081	-2.19	0.029	29.45	19.56		
R^2^ = 0.130, F(9,761) = 12.62, *p* < .001								
**Health history regression**								
Surgery	− 0.050	− 0.031	− 0.88	0.379			723	83.7
Radiation	− 0.089	− 0.073	-2.02	0.044			342	40.3
Chemotherapy	− 0.214	− 0.163	-4.31	0.000			253	29.3
Time since diagnosis	0.024	0.025	0.74	0.459	3.21	0.61		
Disease stage	− 0.021	− 0.033	− 0.88	0.380	1.35	0.95		
Comorbidities	− 0.096	− 0.288	-8.50	0.000	2.12	1.81		
R^2^ = 0.123, F(6,780) = 18 0.20, *p* < .001								
**Health behavior variables regression**								
Current Smoker	− 0.173	− 0.078	-2.13	0.034			62	7.2
Alcohol Use (y/n)	0.141	0.115	3.13	0.002			532	64.1
Physical Activity	0.107	0.160	4.29	0.000	1.18	0.88		
Healthy Diet	0.114	0.132	3.51	0.000	4.46	0.68		
BMI	− 0.004	− 0.040	-1.10	0.274	28.67	6.33		
R^2^ = 0.097, F(5,715) = 15.38, *p* < .001								
**Survivorship care experience regression**								
Provider Survivor Care Practices	− 0.005	− 0.013	− 0.37	0.713	3.72	1.69		
Information Needs	− 0.039	− 0.261	-7.62	0.000	4.27	3.92		
Preparedness for survivorship	0.171	0.227	6.33	0.000	3.28	0.81		
R^2^ = 0.150, F(3,786) = 46.32, *p* < .001								
**Psychosocial variables regression**								
Unmet support needs	− 0.053	− 0.568	-19.45	0.000	4.06	6.36		
Fear of recurrence	− 0.067	− 0.161	-5.53	0.000	2.73	1.45		
R^2^ = 0.424, F(2,812) = 299.03, *p* < .001								

**Note**. Regression coefficients are from the final model that included all predictors. Biological sex 1 = male, 0 = female; White, not Hispanic = 1, Other race/ethnicity = 0; Married = 1, other = 0; BA or more education = 1, less than a BA = 0; Employed 1 = employed, student, homemaker, parttime, 0 = unemployed, retired, disabled; US born = 1, all others = 0; Any Financial hardship was coded 1 = any hardship, 0 = no hardship; Treatment with Surgery, Treatment with Chemotherapy, and Treatment with Radiation were coded 1 = yes, 0 = no; Current Smoker Yes = 1, No = 0; Alcohol Use 1 = Yes in past month 0 = no in past month

**Table** 3 presents the hierarchical regression results predicting overall *HR*QoL with the significant indicators from the specific domains. Regression coefficients and tests are from the full model that included all predictors. Thus, they can be interpreted as the effects of a predictor holding all other variables in the full model constant. The sociodemographic characteristics accounted for 11.4% of the variance in HRQoL. However, the only predictor that was statistically significant holding the other variables constant was employment status, such that employed survivors reported higher HRQoL. The addition of the three health history variables accounted for an additional 5.9% of the HRQoL variance, although only the number of comorbidities was statistically significant. The four health behaviors accounted for 4.0% of the remaining variance, but only a healthier diet was significant controlling for all other variables in the model. The two survivorship care experience variables accounted for 7.7% of the variance in HRQoL over and above sociodemographic, health history, and health behavior variables, with individuals who were higher preparedness for survivorship reporting significantly higher HRQoL controlling for all other variables in the model. Finally, both psychosocial factors were significant predictors of HRQoL. As a set, they accounted for 21.2% of additional variance in HRQoL over and above the other variables in the model, with participants with greater unmet support needs and higher fear of recurrence reporting lower HRQoL.

**Table 3:** Hierarchical Multiple Regression Results predicting overall HRQoL

	*B*		*β*	*t(665)*	*p*
**Sociodemographic characteristics**					
Male sex	0.037		0.031	1.04	0.300
Employed	0.129		0.109	3.78	< 0.001
Financial Hardship	− 0.027		− 0.021	− 0.69	0.491
Area Deprivation Index	0.000		0.011	0.37	0.715
	∆R^2^ = 0.114, F(4,676) = 21.77, p < .001				
**Health history**					
Radiation	− 0.036		− 0.030	-1.04	0.300
Chemotherapy	− 0.035		− 0.026	− 0.90	0.368
Comorbidities	− 0.055		− 0.161	-5.47	< 0.001
	∆R^2^ = 0.059, F(3,673) = 15.95, p < .001				
**Health behaviors**					
Current Smoker	− 0.053		− 0.023	− 0.81	0.421
Alcohol Use in past month (y/n)	0.042		0.033	1.13	0.258
Physical Activity	0.031		0.047	1.62	0.106
Healthy Diet	0.088		0.101	3.41	0.001
	∆R^2^ = 0.040, F(4,669) = 8.54, p < .001				
**Survivorship care experiences**					
Information Needs	0.003		0.020	0.62	0.538
Preparedness for survivorship	0.077		0.102	3.42	< 0.001
	∆R^2^ = 0.077, F(2,667) = 36.16, p < .001				
**Psychosocial factors**					
Unmet support needs	− 0.042		− 0.450	-13.02	< 0.001
Fear of recurrence	− 0.082		− 0.202	-6.27	< 0.001
	∆R^2^ = 0.212, F(2,665) = 141.10, p < .001				

**Note**. Regression coefficients are from the final model that included all predictors. Biological sex was coded 1 = male, 0 = female; Employed was coded 1 = employed, student, homemaker, parttime, 0 = unemployed, retired, disabled; Any Financial hardship was coded 1 = any hardship, 0 = no hardship; Treatment with Chemo and Treatment with Radiation were coded 1 = yes, 0 = no

### Multiple regression results predicting the four subscales of HRQoL

#### Physical well-being

Results from the final model predicting physical well-being are in **Supplemental Table** 1. Unlike overall HRQoL, age was a significant positive predictor of PWB. The set of five sociodemographic predictors accounted for 14.3% of the variance and both employment and age were positive and statistically significant predictors in the full hierarchical model. The same set of health history variables that predicted HRQoL predicted PWB. Both treatment with radiation and comorbidities were significant predictors in the full model, and the health history variables together accounted for 8.5% of the variance. Alcohol use and physical activity were significant positive predictors of physical well-being, and together accounted for 2.6% of the variance. The three survivorship care experience variables accounted for 4.4% of the PWB variance and notably, higher scores on provider survivor care practices were associated with significantly lower physical well-being. Finally, the two psychosocial variables showed significant negative associations with physical well-being and accounted for 18.5% of the variance over and above all other variables in the model.

#### Social well-being

The only sociodemographic variables that predicted SWB (3.3% of the variance; see **Supplemental Table** 2) were marital status and financial hardship, with married individuals reporting higher SWB and those with financial hardships reporting lower SWB, although neither were significant in the full model. Comorbidities was the only health history variable to be included in the full model (2.0% of the variance), such that individuals with more comorbidities reported lower SWB. Individuals who reported healthier diets had higher SWB, although diet only accounted for 0.9% of the variance and was not statistically significant as a predictor in the full model. All three of the survivorship care experience variables were included in the model, together accounting for 5.3% of the variance. In this case, unlike for PWB, the association between provider survivor care practices and SWB was positive, although not statistically significant (*p* = .055). Preparedness for survivorship was a significant positive predictor of SWB. Finally, for psychosocial factors, having more unmet support needs was a significant negative predictor of SWB, accounting for 5.2% of the variance.

#### Emotional well-being

**Supplemental Table** 3 presents the final model predicting EWB. Four sociodemographic characteristics were included in the hierarchical model (biological sex, employment, financial hardship, and age) and although they accounted for 9.0% of the EWB variance, none were predictive of the outcome over and above all the other variables in the model. Treatment with surgery, treatment with chemotherapy, and number of comorbidities accounted for 3.0% of the variance, but only the number of comorbidities was a significant negative predictor of EWB over and above the other variables. Physical activity and healthy diet were included in the model, were both positive but nonsignificant predictors in the final model and accounted for 1.3% of the variance in EWB over and above sociodemographic and health history predictors. Likewise, information needs and preparedness for survivorship were positive but not statistically significant predictors, accounting for 5.4% of the variance. Finally, unmet support needs and fear of recurrence together accounted for 25.2% of the variance in EWB, with both unmet support needs and fear of recurrence showing significant negative associations.

#### Functional well-being

Results for FWB (**Supplemental Table** 4) were similar to those for overall HRQOL in that the same set of health behavior variables, survivorship care experiences, and psychosocial factors predicted functional well-being and HRQoL. The only differences in included variables for the hierarchical model were that biological sex and treatment with radiation were not included as predictors of FWB. Sociodemographic variables accounted for 10.2% of the variance of FWB and employment status was a significant positive predictor when all other variables in the model were taken into account. Both treatment with chemotherapy and number of comorbidities were significant negative predictors of FWB with these two health history variables accounting for 5.5% of the variance. The four health behaviors were included in the hierarchical model (accounting for 4.2% of the variance), and both physical activity and healthy diet were significant positive predictors of FWB. Finally, information needs and preparedness for survivorship together accounted for 3.2% of the variance, but neither was a significant predictor of functional well-being over and above the other variables in the full model. However, both unmet support needs and fear of recurrence were significant negative predictors of FWB, accounting for 17.7% of the variance over and above the other variables.

## Discussion

In this study, we examined the role of social determinants, health history, health behaviors, survivorship care experiences, and psychological factors in the HRQoL in a sample of more than 800 recent cancer survivors residing in New Jersey. Lower overall HRQoL was associated with being unemployed, having more comorbid medical conditions, consuming a less healthy diet, lower preparedness for survivorship, and having more unmet support needs and more fear about cancer recurrence. A number of large studies of cancer survivors have also reported that unemployed survivors experience lower HRQoL, [6, 46, 47] underscoring the important role of optimizing employment outcomes among cancer survivors [40]. Our finding that having more comorbidities is associated with poorer HRQoL is also consistent with other large national studies of survivors’ HRQoL, [2, 29, 48–50] as well as studies conducted in other countries [16, 51]. We did not assess when these comorbid conditions developed, so it is unclear whether these were pre-existing medical conditions that contributed to HRQoL rather than being associated with the cancer diagnosis. Given the average time since diagnosis was approximately three years, it is unlikely that these medical conditions represented late effects of cancer. It is interesting to note that consumption of a less healthy diet was associated with lower overall HRQoL, as well as lower FWB. Other studies of cancer survivors have indicated that improvements in dietary practices (e.g., increases in consumption of fruits, vegetables, fish; decreases in consumption of red meat, sugar) are associated with higher emotional functioning and less fatigue, [52] and other work has also found that healthier diets are associated with higher HRQOL [53–56].

The association between survivorship care experiences and HRQOL has not been well-characterized. Among the factors evaluated, only greater perceived preparedness for survivorship (e.g., satisfaction with information about survivorship, sufficient amount of information provided) was associated with overall HRQoL. Leach and colleagues [57] reported that greater preparedness was associated with lower levels of depressive symptoms, but they did not assess other components of HRQoL. Although we predicted that provider survivorship care practices such as providing a treatment summary, discussing recommended follow-up care, emotional and social needs, and recommended lifestyle changes would be associated with overall HRQoL, they were only associated with the PWB domain of HRQoL. Although Jefford and colleagues [51] found that survivors who stated they had been provided a care plan reported higher HRQoL, a review of the literature on survivorship care plans published in 2018 concluded that the benefit of survivorship care plans on HRQoL was not sufficiently shown from the existing literature [58]. Given the emphasis on models of survivorship care that can best foster the transition to survivorship and higher HRQoL and the potential implications of these findings for services provided by survivorship care transition programs, further evaluation will be important.

Among the factors we assessed, the two psychological factors, unmet support needs and fear of recurrence, played the most important role in HRQoL, accounting for more than 20% of the variance in global HRQoL and both were significant correlates of the physical, functional, and emotional HRQoL domains. Unmet supportive care needs have been consistently associated with lower HRQoL in a few studies focusing on survivors of many types of cancer (e.g., head and neck cancer [50] and breast cancer) [30, 59, 60] but has not been included in large US population survivorship studies [2, 48]. Similarly, fear of recurrence is a known correlate of lower HRQoL in studies of survivors of lymphoma, [61, 62] head and neck cancer, [63] and breast cancer, [64] but has not been included in large US population studies. Although the association with emotional HRQoL is not surprising, the fact that unmet support needs and fears about recurrence were associated with physical and functional domains indicates the important role of cancer-specific emotional responses on HRQoL.

Several factors were not associated with global HRQoL in the final model but were associated with separate domains. Receiving chemotherapy was associated with lower FWB, receiving radiation was associated with lower PWB, and more physical activity was associated with higher physical and FWB. Two counter-intuitive findings were seen: alcohol use was associated with higher PWB and higher survivorship care practices were associated with lower PWB. One explanation for the care practices association is that providers were likely to discuss follow-up care with patients experiencing more physical side effects (e.g., energy, nausea, pain, feeling ill). It is unclear why alcohol use is associated with higher PWB, and this should be examined in future research.

What may be even more important to point out are what factors were *not* associated with HRQoL. Although financial hardship, residing in a high socioeconomically disadvantaged area, smoking, less physical activity, and more information needs were associated with worse overall HRQoL, these associations were attenuated when other factors were included in the model. It is also interesting to note that stage of disease and time since diagnosis were not associated with overall HRQoL or any of the specific domains. It is possible that the relatively low rates of financial hardship, relatively high income, and high number of early-stage cancers in our sample were partially responsible for these findings. Future large survivorship cohort studies would benefit from oversampling more disadvantaged survivor populations and those with later stage disease.

Before concluding, it is important to point out strengths and limitations. In terms of strengths, the use of a state registry provided population-based data that has good external validity. The NJSCR collected basic demographic and medical information from hospital medical records on study refusers (sex, race, ethnicity, age, cancer type, cancer stage) which allowed us to examine of differences between participants and refusers to illuminate potential sources of bias in the sample. In terms of limitations, despite attempts to recruit a diverse population that is representative of the state’s composition, our sample was not as diverse as expected: Our sample was primarily comprised of non-Hispanic whites (75%), which is higher than New Jersey’s profile (51% non-Hispanic White) [65]. Further, 30.8% reported an annual income greater than or equal to $90,000, and 55% completed at least a college-level education. In the future, oversampling socioeconomically disadvantaged and minority survivors would provide information about the state’s more vulnerable survivor population. A second limitation is the cross-sectional design, which makes causal inferences between HRQoL and its correlates difficult. Third, we did not include all cancers in our sample, and future studies should recruit a broader range of cancer types. Fourth, self-report surveys have limitations, particularly when BMI, dietary practices, tobacco use, alcohol use, and physical activity behaviors are being assessed. In addition, self-report measurements correlate with higher with other self-report measures than more objective assessments due to underlying common factors influencing this measurement approach. Future studies could consider using more objective measures. Finally, we examined a broad range of variables as putative correlates of HRQoL, and the findings could be inflated by multiple comparisons.

Despite these limitations, this large cohort of the Rutgers Cancer Institute of New Jersey’s catchment area provides an important resource for characterizing cancer survivors’ HRQoL and those at risk for lower HRQoL. This study illuminates modifiable risk factors such as active and coordinated management of comorbid medical problems, fostering a healthier diet, and assessing and addressing unmet support needs and fears about cancer recurrence may bolster HRQOL, at least among cancer survivors in New Jersey.

## Electronic supplementary material

Below is the link to the electronic supplementary material.

Supplementary Material 1

## Data Availability

The datasets used and/or analyzed during the current study are available from the corresponding author on reasonable request.

## Abbreviations

HRQoL: Health-related quality of life
PWB: Physical well-being
SWB: Social/family well-being
EWB: Emotional well-being
FWB: Functional well-being
unmet needs: Fear of recurrence

## References

[1] Cancer Stats and Figures. Atlanta, American Cancer Society. : ; 2022. Available from: https://www.cancer.org/research/cancer-facts-statistics/all-cancer-facts-figures/cancer-facts-figures-2022.html.

[2] Han X, Robinson LA, Jensen RE, Smith TG, Yabroff KR. Factors Associated with Health-Related Quality of Life among Cancer Survivors in the United States. JNCI Cancer Spectr. 2021;5(1). 10.1093/jncics/pkaa123 PMC7883550 33615136

[3] Richardson LC, Wingo PA, Zack MM, Zahran HS, King JB (2008). Health-related quality of life in cancer survivors between ages 20 and 64 years: population-based estimates from the behavioral risk factor Surveillance System. Cancer.

[4] Weaver KE, Forsythe LP, Reeve BB, Alfano CM, Rodriguez JL, Sabatino SA et al. Mental and physical health-related quality of life among U.S. cancer survivors: population estimates from the 2010 National Health interview survey. Cancer epidemiology, biomarkers & prevention : a publication of the American Association for Cancer Research, cosponsored by the American Society of Preventive Oncology. 2012;21(11):2108–17. 10.1158/1055-9965.EPI-12-0740 PMC3645867 23112268

[5] Sharma N, Purkayastha A (2017). Factors affecting quality of life in breast Cancer patients: a descriptive and cross-sectional study with review of literature. J Midlife Health.

[6] Andreu Y, Martínez P, Soto-Rubio A, Pérez-Marín M, Cervantes A, Arribas L. Quality of life in cancer survivorship: Sociodemographic and disease-related moderators. Eur J Cancer Care (Engl). 2022:e13692. 10.1111/ecc.13692 36069249

[7] Parker PA, Baile WF, de Moor C, Cohen L (2003). Psychosocial and demographic predictors of quality of life in a large sample of cancer patients. Psychooncology.

[8] Giedzinska AS, Meyerowitz BE, Ganz PA, Rowland JH (2004). Health-related quality of life in a multiethnic sample of breast cancer survivors. Ann Behav Med.

[9] Matthews AK, Tejeda S, Johnson TP, Berbaum ML, Manfredi C (2012). Correlates of quality of life among african american and white cancer survivors. Cancer Nurs.

[10] Belachew AA, Reyes ME, Ye Y, Raju GS, Rodriguez MA, Wu X (2020). Patterns of racial/ethnic disparities in baseline health-related quality of life and relationship with overall survival in patients with colorectal cancer. Qual Life Res.

[11] Hastert TA, Kyko JM, Reed AR, Harper FWK, Beebe-Dimmer JL, Baird TE (2019). Financial hardship and quality of life among african american and White Cancer Survivors: the role of limiting Care due to cost. Cancer epidemiology, biomarkers & prevention : a publication of the American Association for Cancer Research. cosponsored by the American Society of Preventive Oncology.

[12] Ver Hoeve ES, Ali-Akbarian L, Price SN, Lothfi NM, Hamann HA (2021). Patient-reported financial toxicity, quality of life, and health behaviors in insured US cancer survivors. Support Care Cancer.

[13] Paskett ED, Alfano CM, Davidson MA, Andersen BL, Naughton MJ, Sherman A (2008). Breast cancer survivors’ health-related quality of life : racial differences and comparisons with noncancer controls. Cancer.

[14] Bowen DJ, Alfano CM, McGregor BA, Kuniyuki A, Bernstein L, Meeske K (2007). Possible socioeconomic and ethnic disparities in quality of life in a cohort of breast cancer survivors. Breast Cancer Res Treat.

[15] Siembida EJ, Smith AW, Potosky AL, Graves KD, Jensen RE (2021). Examination of individual and multiple comorbid conditions and health-related quality of life in older cancer survivors. Qual Life Res.

[16] Götze H, Taubenheim S, Dietz A, Lordick F, Mehnert A (2018). Comorbid conditions and health-related quality of life in long-term cancer survivors-associations with demographic and medical characteristics. J Cancer Surviv.

[17] Anbari AB, Deroche CB, Armer JM (2019). Body mass index trends and quality of life from breast cancer diagnosis through seven years’ survivorship. World J Clin Oncol.

[18] Grimmett C, Bridgewater J, Steptoe A, Wardle J (2011). Lifestyle and quality of life in colorectal cancer survivors. Qual Life Res.

[19] Breedveld-Peters JJL, Koole JL, van der Müller-Schulte E, Windhausen C, Bours MJL (2018). Colorectal cancers survivors’ adherence to lifestyle recommendations and cross-sectional associations with health-related quality of life. Br J Nutr.

[20] Mosher CE, Sloane R, Morey MC, Snyder DC, Cohen HJ, Miller PE (2009). Associations between lifestyle factors and quality of life among older long-term breast, prostate, and colorectal cancer survivors. Cancer.

[21] Pophali PA, Larson MC, Rosenthal AC, Robinson D, Habermann TM, Thanarajasingam G (2021). The association of health behaviors with quality of life in lymphoma survivors. Leuk Lymphoma.

[22] Aarstad AK, Aarstad HJ, Olofsson J (2007). Quality of life, drinking to cope, alcohol consumption and smoking in successfully treated HNSCC patients. Acta Otolaryngol.

[23] McCarter K, Baker AL, Britton B, Wolfenden L, Wratten C, Bauer J (2018). Smoking, drinking, and depression: comorbidity in head and neck cancer patients undergoing radiotherapy. Cancer Med.

[24] Dahl AA, Haaland CF, Mykletun A, Bremnes R, Dahl O, Klepp O (2005). Study of anxiety disorder and depression in long-term survivors of testicular cancer. J Clin Oncol.

[25] Révész D, Bours MJL, Wegdam JA, Keulen ETP, Breukink SO, Slooter GD (2022). Associations between alcohol consumption and anxiety, depression, and health-related quality of life in colorectal cancer survivors. J Cancer Surviv.

[26] Chung J, Kulkarni GS, Morash R, Matthew A, Papadakos J, Breau RH (2019). Assessment of quality of life, information, and supportive care needs in patients with muscle and non-muscle invasive bladder cancer across the illness trajectory. Support Care Cancer.

[27] Handschel J, Naujoks C, Kübler NR, Krüskemper G (2012). Fear of recurrence significantly influences quality of life in oral cancer patients. Oral Oncol.

[28] Abu Sharour L, Malak M, Subih M, Bani Salameh A (2020). Quality of life, care needs, and information needs among patients diagnosed with cancer during their treatment phase. Psychol health Med.

[29] Oberoi DV, White VM, Seymour JF, Prince HM, Harrison S, Jefford M (2017). Distress and unmet needs during treatment and quality of life in early cancer survivorship: a longitudinal study of haematological cancer patients. Eur J Haematol.

[30] Pongthavornkamol K, Lekdamrongkul P, Pinsuntorn P, Molassiotis A, Physical, Symptoms (2019). Unmet needs, and Quality of Life in Thai Cancer Survivors after the completion of primary treatment. Asia Pac J Oncol Nurs.

[31] Llanos AAM, Fong AJ, Ghosh N, Devine KA, O’Malley D, Paddock LE et al. COVID-19 perceptions, impacts, and experiences: A cross-sectional analysis among New Jersey cancer survivors. Journal of Cancer Survivorship. In Press. 10.1007/s11764-022-01236-6 PMC9336177 35904727

[32] Cella DF, Tulsky DS, Gray G, Sarafian B, Linn E, Bonomi A (1993). The Functional Assessment of Cancer Therapy scale: development and validation of the general measure. J Clin Oncol.

[33] Cancer Experiences Questionnaire: Agency for Healthcare Research and Quality; 2017 [Available from: https://meps.ahrq.gov/survey_comp/hc_survey/paper_quest/2017/CancerSAQ_En.shtml.

[34] Beckjord EB, Arora NK, McLaughlin W, Oakley-Girvan I, Hamilton AS, Hesse BW (2008). Health-related information needs in a large and diverse sample of adult cancer survivors: implications for cancer care. J Cancer Surviv.

[35] Singh GK (2003). Area deprivation and widening inequalities in US mortality, 1969–1998. Am J Public Health.

[36] Kind AJ, Jencks S, Brock J, Yu M, Bartels C, Ehlenbach W (2014). Neighborhood socioeconomic disadvantage and 30-day rehospitalization: a retrospective cohort study. Ann Intern Med.

[37] Kind AJH, Buckingham WR (2018). Making Neighborhood-Disadvantage Metrics Accessible - The Neighborhood Atlas. N Engl J Med.

[38] Cantor D, Coa K, Crystal-Mansour S, Davis T, Dipko S, Sigman R. Health Information Trends Survey (HINTS) 2007: Final Report. 2007.

[39] Nelson DE, Kreps GL, Hesse BW, Croyle RT, Willis G, Arora NK (2004). The Health Information National Trends Survey (HINTS): development; design, and dissemination. J Health Communication.

[40] Follow-up Care Use Among Survivors (FOCUS) Survey. : National Cancer Institute, Division of Cancer Control & Population Sciences; [updated September 24 2020; cited 2022 February 2]. Available from: https://cancercontrol.cancer.gov/ocs/resources/researchers/FOCUS.

[41] Godin G (2011). The Godin-Shephard leisure-time physical activity questionnaire. The Health & Fitness Journal of Canada.

[42] Diet N, World Cancer Research Fund International. Physical Activity and Cancer: a Global Perspective Pentonville Road, London: ; 2018 [Available from: https://www.wcrf.org/diet-activity-and-cancer/global-cancer-update-programme/resources-and-toolkits/.

[43] Cantor D, Coa K, Crystal-Mansour S, Davis T, Dipko S, sigman R. Health Information Trends Survey (HINTS) 2007: Final Report2009. Available from: https://hints.cancer.gov/docs/HINTS2007FinalReport.pdf.

[44] Manne SL, Smith BL, Frederick S, Mitarotondo A, Kashy DA, Kirstein LJ (2020). B-Sure: a randomized pilot trial of an interactive web-based decision support aid versus usual care in average-risk breast cancer patients considering contralateral prophylactic mastectomy. Transl Behav Med.

[45] Campbell HS, Sanson-Fisher R, Turner D, Hayward L, Wang XS, Taylor-Brown J (2010). Psychometric properties of cancer survivors’ unmet needs survey. Support Care Cancer.

[46] de Souza JA, Yap BJ, Wroblewski K, Blinder V, Araújo FS, Hlubocky FJ (2017). Measuring financial toxicity as a clinically relevant patient-reported outcome: the validation of the COmprehensive score for financial toxicity (COST). Cancer.

[47] Mehnert A (2011). Employment and work-related issues in cancer survivors. Crit Rev Oncol Hematol.

[48] Huang IC, Hudson MM, Robison LL, Krull KR. Differential Impact of Symptom Prevalence and Chronic Conditions on Quality of Life in Cancer Survivors and Non-Cancer individuals: a Population Study. Cancer epidemiology, biomarkers & prevention : a publication of the American Association for Cancer Research, cosponsored by the American Society of Preventive Oncology. 2017;26(7):1124–32. 10.1158/1055-9965.EPI-16-1007 PMC5500424 28336581

[49] Davies LM, Hayhurst KP, Lorigan P, Molassiotis A (2018). Unmet supportive care needs, health status and minimum costs in survivors of malignant melanoma. Eur J Cancer Care (Engl).

[50] So WK, Choi KC, Chen JM, Chan CW, Chair SY, Fung OW (2014). Quality of life in head and neck cancer survivors at 1 year after treatment: the mediating role of unmet supportive care needs. Support Care Cancer.

[51] Jefford M, Ward AC, Lisy K, Lacey K, Emery JD, Glaser AW (2017). Patient-reported outcomes in cancer survivors: a population-wide cross-sectional study. Support Care Cancer.

[52] Zainordin NH, Abd Talib R, Shahril MR, Sulaiman S (2020). Dietary changes and its impact on quality of life among malay breast and Gynaecological Cancer Survivors in Malaysia. Asian Pac J Cancer Prev.

[53] Blanchard CM, Courneya KS, Stein K (2008). Cancer survivors’ adherence to lifestyle behavior recommendations and associations with health-related quality of life: results from the American Cancer Society’s SCS-II. J Clin Oncol.

[54] Krok-Schoen JL, Pisegna J, Arthur E, Ridgway E, Stephens C, Rosko AE (2021). Prevalence of lifestyle behaviors and associations with health-related quality of life among older female cancer survivors. Support Care Cancer.

[55] Mohammadi S, Sulaiman S, Koon PB, Amani R, Hosseini SM (2013). Impact of healthy eating practices and physical activity on quality of life among breast cancer survivors. Asian Pac J Cancer Prev.

[56] Rim CH, Ahn SJ, Kim JH, Yoon WS, Chun M, Yang DS (2019). Questionnaire study of the dietary habits of breast cancer survivors and their relationship to quality of life (KROG 14 – 09). Eur J Cancer Care (Engl).

[57] Leach CR, Troeschel AN, Wiatrek D, Stanton AL, Diefenbach M, Stein KD (2017). Preparedness and Cancer-related Symptom Management among Cancer Survivors in the First Year Post-Treatment. Ann Behav Med.

[58] Jacobsen PB, DeRosa AP, Henderson TO, Mayer DK, Moskowitz CS, Paskett ED (2018). Systematic review of the impact of Cancer Survivorship Care Plans on Health Outcomes and Health Care Delivery. J Clin Oncol.

[59] Cheng KKF, Wong WH, Koh C (2016). Unmet needs mediate the relationship between symptoms and quality of life in breast cancer survivors. Support Care Cancer.

[60] Faller H, Brähler E, Härter M, Keller M, Schulz H, Wegscheider K (2017). Unmet needs for information and psychosocial support in relation to quality of life and emotional distress: a comparison between gynecological and breast cancer patients. Patient Educ Couns.

[61] Ellis GK, Chapman H, Manda A, Salima A, Itimu S, Banda G (2021). Pediatric lymphoma patients in Malawi present with poor health-related quality of life at diagnosis and improve throughout treatment and follow-up across all Pediatric PROMIS-25 domains. Pediatr Blood Cancer.

[62] Ellis S, Brown RF, Thorsteinsson EB, Pakenham KI, Perrott C (2022). Quality of life and fear of cancer recurrence in patients and survivors of non-hodgkin lymphoma. Psychol health Med.

[63] Tsay SL, Wang JY, Lee YH, Chen YJ (2020). Fear of recurrence: a mediator of the relationship between physical symptoms and quality of life in head and neck cancer patients. Eur J Cancer Care (Engl).

[64] Tran TXM, Jung SY, Lee EG, Cho H, Kim NY, Shim S (2022). Fear of Cancer recurrence and its negative impact on Health-Related Quality of Life in Long-term breast Cancer survivors. Cancer Res Treat.

[65] Hispanic or Latino., and not Hispanic or Latino by Race: United States Census Bureau; 2020 [Available from: https://data.census.gov/cedsci/table?g=0400000US34&tid=DECENNIALPL2020.P2.

