# Supplementary material for: Factors associated with health-related quality of life in a cohort of cancer survivors in New Jersey
Source: BMC Cancer. 2023 Jul 14;23:664. doi: 10.1186/s12885-023-11098-5 (PMC10349446; doi:10.1186/s12885-023-11098-5)
Supplement: Supplementary file 1 — Supplementary Material 1 [file 12885_2023_11098_MOESM1_ESM.docx]

**Supplemental Table 1. Hierarchical Multiple Regression Results Predicting**

**Physical Well-Being**

|  | ***b*** | ***β*** | ***t(700)*** | ***P*** |
| --- | --- | --- | --- | --- |
| **Social determinants** |  |  |  |  |
| Biological Sex: Male | .039 | .029 | .99 | .322 |
| Employed | .140 | .106 | 3.17 | .002 |
| Financial Hardship | -.045 | -.032 | -1.03 | .301 |
| Age | .006 | .090 | 2.53 | .011 |
| Area Deprivation Index | -.001 | -.023 | -.80 | .427 |
| ∆R^2^ =.143, F(5,710) = 23.71, p < .001 | | | | |
| **Health history** |  |  |  |  |
| Radiation | -.112 | -.084 | -2.91 | .004 |
| Chemotherapy | -.060 | -.041 | -1.41 | .159 |
| Comorbidities | -.061 | -.165 | -5.62 | .000 |
| ∆R^2^ =.085, F(3,707) = 25.80, p < .001 | | | | |
| **Health behaviors** |  |  |  |  |
| Alcohol Use in past month | .097 | .070 | 2.42 | .016 |
| Physical Activity | .074 | .100 | 3.51 | .000 |
| ∆R^2^ =.026, F(2,705) = 12.23, p = .001 | | | | |
| **Survivorship care experiences** |  |  |  |  |
| Survivorship Care Practices | -.036 | -.089 | -2.85 | .004 |
| Information Needs | .002 | .011 | .35 | .724 |
| Preparedness for survivorship | .043 | .051 | 1.63 | .104 |
| ∆R^2^ =.044, F(3,702) = 14.50, p < .001 | | | | |
| **Psychosocial factors** |  |  |  |  |
| Unmet support needs | -.049 | -.479 | -13.81 | .000 |
| Fear of recurrence | -.047 | -.104 | -3.22 | .001 |
| ∆R^2^ =.185, F(2,700) = 125.12, p < .001 | | | | |

**Note**. Regression coefficients are from the final model that included all predictors.

**Supplemental Table 2. Hierarchical Multiple Regression Results**

**Predicting Social Well-Being**

|  | ***b*** | ***β*** | ***t(755)*** | ***p*** |
| --- | --- | --- | --- | --- |
| **Social determinants** |  |  |  |  |
| Married | .088 | .048 | 1.41 | .160 |
| Financial Hardship | -.057 | -.031 | -.85 | .396 |
| ∆R^2^ =.033, F(2,761) = 12.98*, p* < .001 | | | | |
| **Health history** |  |  |  |  |
| Comorbidities | -.054 | -.110 | -3.26 | .001 |
| ∆R^2^ =.020, F(1,760) = 16.42, *p* < .001 | | | | |
| **Health behaviors** |  |  |  |  |
| Healthy Diet | .073 | .058 | 1.69 | .092 |
| ∆R^2^ =.009, F(1,759) = 6.96, *p* = .008 | | | | |
| **Survivorship care experiences** |  |  |  |  |
| Survivorship Care Practices | .035 | .067 | 1.92 | .055 |
| Information Needs | -.003 | -.012 | -.33 | .741 |
| Preparedness for survivorship | .109 | .103 | 2.78 | .006 |
| ∆R^2^ =.053, F(3,756) = 15.10, *p* < .001 | | | | |
| **Psychosocial factors** |  |  |  |  |
| Unmet support needs | -.037 | -.273 | -6.86 | .000 |
| ∆R^2^ =.052, F(1,755) = 46.99, *p* < .001 | | | | |

**Note**. Regression coefficients are from the final model that included all predictors.

**Supplemental Table 3. Hierarchical Multiple Regression Results**

**Predicting Emotional Well-Being**

|  | ***b*** | ***β*** | ***t(711)*** | ***P*** |
| --- | --- | --- | --- | --- |
| **Social determinants** |  |  |  |  |
| Biological Sex: Male | .052 | .047 | 1.47 | .143 |
| Employed | .032 | .029 | .84 | .402 |
| Any Financial Hardship | -.019 | -.016 | -.53 | .599 |
| Age | .000 | .006 | .17 | .868 |
| ∆R^2^ =.090, F(4,720) = 17.79, *p* < .001 | | | | |
| **Health history** |  |  |  |  |
| Surgery | .017 | .011 | .36 | .721 |
| Chemotherapy | .005 | .004 | .13 | .897 |
| Comorbidities | -.028 | -.092 | -3.07 | .002 |
| ∆R^2^ =.030, F(3,717) = 8.12, *p* < .001 | | | | |
| **Health behaviors** |  |  |  |  |
| Physical Activity | .006 | .010 | .33 | .743 |
| Healthy Diet | .028 | .035 | 1.19 | .234 |
| ∆R^2^ =.013, F(2,715) = 5.20, *p* =.006 | | | | |
| **Survivorship care experiences** |  |  |  |  |
| Information Needs | .003 | .025 | .76 | .448 |
| Preparedness for survivorship | .037 | .054 | 1.76 | .078 |
| ∆R^2^ =.054, F(2,713) = 23.62, *p* < .001 | | | | |
| **Psychosocial factors** |  |  |  |  |
| Unmet support needs | -.035 | -.403 | -11.45 | .000 |
| Fear of recurrence | -.122 | -.326 | -9.81 | .000 |
| ∆R^2^ =.252, F(2,711) = 159.35, *p* < .001 | | | | |

**Note**. Regression coefficients are from the final model that included all predictors.

**Supplemental Table 4. Hierarchical Multiple Regression Results**

**Predicting Functional Well-Being**

|  | ***b*** | ***β*** | ***t(691)*** | ***p*** |
| --- | --- | --- | --- | --- |
| **Social determinants** |  |  |  |  |
| Employed | .369 | .198 | 6.31 | .000 |
| Financial Hardship | .014 | .007 | .20 | .840 |
| Area Deprivation Index | .000 | -.006 | -.19 | .851 |
| ∆R^2^ =.102, F(3,701) = 26.61, *p* < .001 | | | | |
| **Health history** |  |  |  |  |
| Chemotherapy | -.137 | -.066 | -2.10 | .036 |
| Comorbidities | -.079 | -.148 | -4.60 | .000 |
| ∆R^2^ =.055, F(2,699) = 22.78, *p* < .001 | | | | |
| **Health behaviors** |  |  |  |  |
| Current smoker | -.132 | -.037 | -1.15 | .251 |
| Alcohol Use in past month | .038 | .020 | .61 | .544 |
| Physical Activity | .067 | .064 | 2.02 | .044 |
| Healthy Diet | .166 | .122 | 3.82 | .000 |
| ∆R^2^ =.042, F(4,695) = 9.22, *p* < .001 | | | | |
| **Survivorship care experiences** |  |  |  |  |
| Information Needs | .014 | .059 | 1.68 | .094 |
| Preparedness for survivorship | .073 | .061 | 1.90 | .058 |
| ∆R^2^ =.032, F(2,693) = 14.58, p < .001 | | | | |
| **Psychosocial factors** |  |  |  |  |
| Unmet support needs | -.052 | -.348 | -9.19 | .000 |
| Fear of recurrence | -.125 | -.195 | -5.62 | .000 |
| ∆R^2^ =.147, F(2,691) = 81.47, p < .001 | | | | |

**Note**. Regression coefficients are from the final model that included all predictors.
